# The emission and physicochemical properties of airborne microplastics and nanoplastics generated during the mechanical recycling of plastic via shredding

**DOI:** 10.1038/s41598-024-73775-0

**Published:** 2024-10-21

**Authors:** S. Swinnerton, J. Su, Candace S. J. Tsai

**Affiliations:** 1 Department of Environmental Health Sciences, Fielding School of Public Health, University of California Los Angeles (UCLA), 650 Charles E. Young Drive S., MC 177220, Los Angeles, CA USA; 2 California NanoSystems Institute, University of California Los Angeles, Los Angeles, CA USA

**Keywords:** Environmental sciences, Health occupations, Nanoscience and technology

## Abstract

This study examined the emission and physicochemical properties of microplastics and nanoplastics (MPs/NPs) generated during shredding, which is regularly used in mechanical recycling. Waste and new polyethylene terephthalate, polypropylene, and high-density polyethylene were investigated herein for a total of six categories. The concentration and size distribution of particles were measured using two spectrometer instruments, and morphology and elemental composition of emitted particles were analyzed with microscopy and spectroscopy. This study found that number concentrations in both submicron and micron sizes of respirable particles were 3–2910× higher during periods of shredding than pre-shredding background concentrations. Maximum concentrations of particles within 10–420 nm, across all six categories, ranged from 22,000- to 1,300,000-particles/cm^3^ during shredding, compared to average background levels of 700 particles/cm^3^. Maximum concentrations of particles within 0.3 to 10 μm, across all six categories, ranged from 24- to 2000-particles/cm^3^ during shredding, compared to average background levels of 2 particles/cm^3^. Waste plastics consistently generated higher emissions than their new counterparts, which is attributed to the labels, adhesives, and increased additives incorporated into the waste plastic. Morphology varied drastically between particles and an elemental composition analysis found that the samples consisted primarily of C and O, representing the polymer material, as well as Na, Mg, Al, Si, Cu, Cl, K, Ca, Ti, Fe, Rb, and Br representing additives, label, and other contaminates. The shredding of plastic has the potential to expose workers to elevated concentrations of airborne MPs/NPs, especially those between 10 and 100 nm.

## Introduction

As the production of plastic has skyrocketed in the past century, the management of plastic waste has become an ongoing challenge. Only 9% of global plastics are recycled while 12% are incinerated and 79% are dumped in landfills or the natural environment^1^. Of the 400 million tons of plastic that are produced annually, 19–23 million tons enter marine ecosystems^2^. The world is experiencing a pollution crisis, one that is demanding a solution where recycling will play a major role. Mechanical recycling, the most common method for plastics, recovers plastic waste via shredding and regranulation and is expected to process almost 55 million tons of plastic globally by 2030^3,4^. Plastic shredding is used in several industries in addition to recycling, including injection molding and extrusion plants, post-consumer plastic waste management, automotive and electronics industries, and medical and pharmaceutical waste disposal. Less established industries also use plastic shredders, such as the emerging process of using plastic waste in construction bricks. Plastic-waste bricks are manufactured throughout developing countries in small-scale facilities that lack proper control measures, resulting in potential occupational health and environmental risks arising from shredding that have yet to be explored. It was also found that research on the emissions associated with plastic shredding in general is absent in existing scientific literature. Thus, in order to address this gap and examine shredding as a potential hazard to users, this study investigates the emission and physicochemical properties of microplastics and nanoplastics (MPs/NPs) generated during shredding.

The physicochemical properties of MPs/NPs drive the toxic mechanisms in which the particles interact with living organisms. Physical characteristics like particle size, morphology, surface charge, zeta potential, and hydrophobicity are all factors that may affect the cellular uptake of a particle and, consequently, shape the particle’s capacity to exert adverse impacts on human health^5–7^. Other physical properties of MPs that can affect toxicity are surface ligands, aging, and roughness^7^. The chemical composition of plastics also plays a key role in dictating the risk of MP/NP exposure. MPs/NPs are able to absorb other chemicals, thus resulting in combined toxicities^8^. Absorbed chemicals, either incorporated during the manufacturing process or adsorbed from the environment, make the threat of MPs/NPs increasingly complex and variable^8,9^. During production, plastics are commonly combined with other compounds, such as inorganic additives like silica to improve physical strength and integrity, inorganic pigments like Fe and Cd to produce red and yellow colors, and antioxidants like Al and Si to prevent degradation^10^. MPs/NPs from waste plastics can also load various environmental pollutants like heavy metals, polycyclic aromatic hydrocarbons, and pesticides via surface adsorption. The risk associated with exposure to MPs/NPs relies heavily on these physicochemical properties, and existing research concludes that no inferences can be made about the effects of MPs/NPs—not even for particles of the same size and polymer—unless their physicochemical properties are well understood^11^. As a result, characterizing the properties of MPs/NPs generated during shredding, in addition to emission concentrations, allows insight on inhalation exposure to users during recycling activities.

It has long been established that particle deposition in the respiratory tract after inhalation is affected by particle size, shape, and charge^12^. Consequently, the health impacts resulting from exposure to MPs/NPs are dependent on such properties^10^. Larger particles inhaled into the lungs tend to settle in the upper respiratory system, while smaller particles are able to reach the alveoli and the cross into the bloodstream^7^. Particles larger than 3–4 microns were found to have decreased alveolar depositions^13,14^. On the other hand, deposition in all regions of the lung were found to increase with decreasing particle diameter below 0.5 μm, with particles between 10 and 30 nm diameter having the highest deposition fraction^15–17^. Particle shape also affects particle behavior and outcome in the lungs. For example, pollen-shaped particles have been found to exhibit better flowability, aerosolization, and deposition properties after inhalation compared to other particle shapes, including spheres, needles, cubes, and plates^18^. Other research suggests that rod-like particles with larger aspect ratios can reach the deeper aspects of the respiratory system^19^. Lastly, surface chemistry affects particle deposition. While the deposition due to charge is usually small compared to deposition by mechanical mechanisms, researchers found that particle deposition enhanced by increasing the level of charge applied to 1 μm aerosol particles^20^. Particle charge and zeta potential are also indicative of a particle’s tendency to agglomerate. One study states that, although small, charged particles may have increased aggregation, there is probably a small effect upon total deposition in the respiratory tract^21^. However, other research finds that small particles that agglomerate can become more inertial, which, if large enough, can change particle trajectory or chance of rebound and in turn cause early deposition^22^. Regardless, if the agglomeration of particles is large enough, it will have the same effect as particle size and settle in the upper respiratory tract^23^.

Shredding activities generate airborne particulate matter that has yet to be investigated in scientific research. The objectives of this study were to explore the magnitude of emission and physicochemical properties of airborne particles generated by shredding to assess occupational inhalation exposure. We evaluated three of the most common types of recycled plastic, polyethylene terephthalate (PET), polypropylene (PP), and high-density polyethylene (HDPE), to simulate mechanical recycling facilities. Furthermore, both waste and new products were evaluated for each polymer type, for a total of six plastic categories, to determine how the emissions vary throughout a product’s life cycle. The physicochemical properties investigated herein include size, morphology, zeta potential, and elemental composition. Defining such properties will allow the characterization of particles and provide an understanding of the potential risk shredding activities pose to human health.

## Results

### Airborne particles generated during shredding

Changes in the total particle concentration were observed during periods of shredding while the background and post-shredding concentrations were relatively consistent. As shown in Table 1, the maximum and average concentrations observed by the NanoScan Scanning Mobility Sizer (SMPS, 10–420 nm) during the periods of shredding ranged from approximately 22,000- to 1,300,000-particles per cubic centimeter (particles/cm^3^) and 6500- to 470,000-particles/cm^3^, respectively. The maximum and average concentrations observed by the Optical Particle Sizer (OPS, 0.3–10 μm) during the periods of shredding ranged from approximately 24- to 2000-particles/cm^3^ and 8- to 470- particles/cm^3^, respectively. This study found that number concentrations of particles were 3 to 2910× higher during periods of shredding than pre-shredding background concentrations. The average background levels measured by the SMPS were approximately 700 particles/cm^3^, while the average background levels measured by the OPS were 2 particles/cm^3^.

**Table 1:** SMPS and OPS total particle number concentration maximums, averages, and standard deviations for each category of plastic.

Plastic type		SMPS (particle/cm^3^)			OPS (particle/cm^3^)		
		Maximum	Average	Standard deviation	Maximum	Average	Standard deviation
Waste	PET	200,000	80,000	45,000	1700	470	230
	PP	1,300,000	470,000	300,000	190	59	32
	HDPE	56,000	12,000	8500	2000	340	300
New	PET	57,000	32,000	11,000	39	21	4
	PP	74,000	59,000	15,000	84	42	8
	HDPE	22,000	6500	4900	24	8	3

**Fig. 1:**
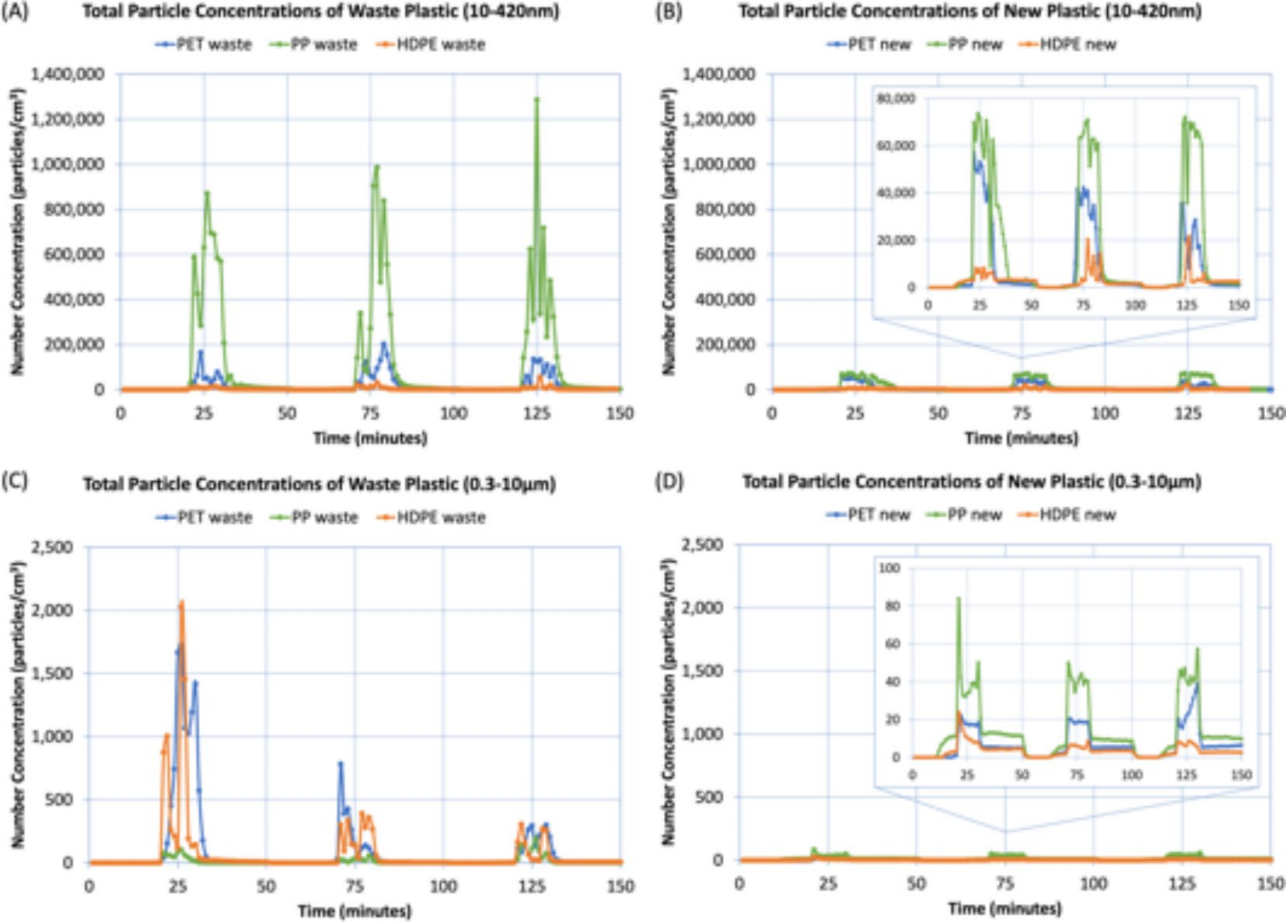
Total particle concentrations during each day of sampling, as measured by the SMPS for particles in the 10–420 nm size range and OPS for particles in the 0.3–10 μm. The figure is organized as follows: (A) SMPS data for waste plastics, (B) SMPS data for new plastics, (C) OPS data for waste plastics, and (D) OPS data for new plastics.

Figure 1 contains graphs of the total concentrations of airborne particles within a 10–420 nm size range, as measured by the SMPS (A, B), and particles within a 0.3–10 μm size range, as measured by the OPS (C, D). The peaks observed in each of the graphs correlate to the three periods of shredding. Waste plastics (A, C) and new plastics (B, D) are presented separately in Fig. 1.

The order of highest to lowest total particle number concentrations measured by the SMPS is as follows: PP waste > PET waste > PP new > PET new > HDPE waste > HDPE new. The average concentration generated by PP waste was almost 5× that of PET waste, which was the plastic with the next highest emissions. Two important conclusions can be drawn: (1) for plastics of both waste and new origins, the emissions generated from each type of plastic adhered to the following order: PP > PET > HDPE, and (2) the waste plastics had higher emissions than their new counterparts, which we attribute to the labels, adhesives, color, and increased additives incorporated into the waste plastic.

In regard to the OPS data, the shredding of waste plastics again generated higher emissions than the new plastics. For the new plastics, the order of highest to lowest total particle number concentrations measured by the OPS was also consistent with the SMPS data: PP > PET > HDPE. However, the concentrations observed during the shredding of waste plastics differed from the previously described observations. For example, PP waste had the lowest particle concentrations compared to PET and HDPE waste. Moreover, while the emissions generated by the PP waste were consistently low (less than 200 particles/cm^3^) during all three experiments, the shredding of PET and HDPE waste had exceptionally high concentrations in the first experiment. To ensure these reported values were not outliers, multiple shredding experiments were conducted, and similar results were observed. Regardless, while the emissions measured by the OPS for waste plastics were an anomaly, it still supported the general finding that the shredding waste plastics resulted in higher emissions than new, unused plastics.

In attempts to explain the observed emission concentrations, studies evaluating the mechanical properties of the different types of plastic were explored. A majority of studies assess the strength and mechanical characteristics of polymer blends (i.e. PET, PP, and HDPE mixed together) and plastic composites with other materials, which are not applicable to this study^24–26^. The mechanical properties of plastic vary drastically based on the percentage of polymer and the addition of additives and fillers, resulting in inconsistent data and no general consensus^27,28^. For example, one study ranked the hardness and elastic modulus of each plastic as follows: PET > HDPE > PP^29^. On the other hand, the hardness of recycled PP has been found to have a slightly higher than recycled HDPE. Though, in the same study, impact resistance was observed to be larger in HDPE^26^. Due to the unknown additive compositions, and large variation of plastic waste products used in this study, no conclusions were found on whether differences in the mechanical properties of the six categories of plastic correlate to the emissions observed during shredding.

Lastly, the mass concentrations, calculated using the change in mass on the filter samples, sampling duration, and the designated flow rate, were all non-quantifiable. This is attributed to the fact that the high number of MP/NP particles, especially those in the submicron size range, have masses too low to be measurable when these particles were collected for a total of 30 min of shredding. The remainder of the approximate 2.5-hour duration was in the cleanroom with very minimal, if any, existing background particulates. However, the high number counts of emitted particles will contribute to a measurable mass when operators perform the shredding activity for a longer period of time in a work shift.

### Physicochemical characteristics of particles released during shredding

#### Size and morphology

##### Particle size fractioned concentrations

**Fig. 2:**
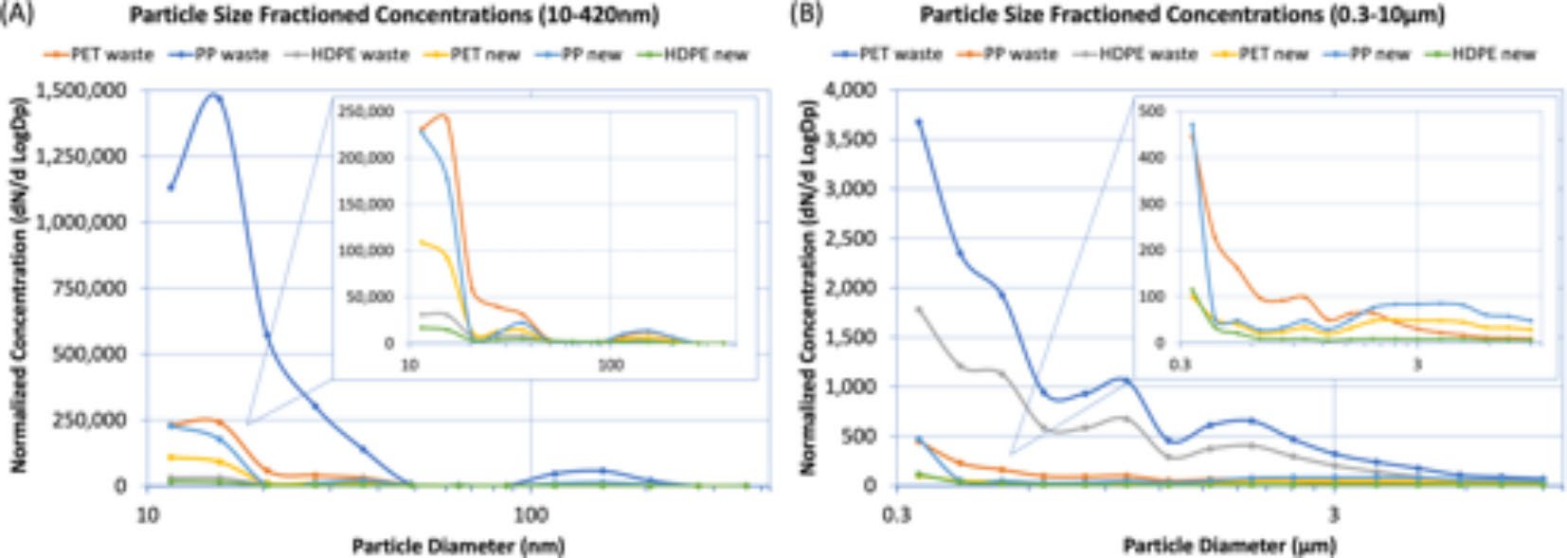
Particle size fractioned concentrations for the six categories of plastic as measured by the (A) SMPS and (B) OPS. The X-axis, particle diameter, is on a logarithmic scale.

Size differentiated graphs of the normalized particle concentrations measured by the SMPS and OPS are presented in Fig. 2. The concentrations are presented in a normalized scale in dN/dlogDp (dN is the number of particles in the range and dlogDp is the difference in the log of the channel width) and are averages of the data points collected during all three experiments for each plastic. The results demonstrate that there were high levels of nanoparticles regardless of plastic type.

In regard to the 10–420 nm particles quantified by the SMPS, PET waste and PP waste had the highest particle concentrations at a diameter midpoint of 15.4 nm. HDPE waste, PET new, PP new, and HDPE new had the highest particle concentrations at a diameter midpoint of 11.5 nm. Particle concentrations mostly decreased after their respective maximums. However, around the 100 nm size range, there was an increase in particle concentrations between diameter midpoint 86.6 and 115.5 nm (PET waste: +7600 particles/cm^3^; PP waste: +43,000 particles/cm^3^; HDPE waste: +1400 particles/cm^3^; PET new: +4200 particles/cm^3^; PP new: +9600 particles/cm^3^; HDPE new: +110 particles/cm^3^). Interestingly, only the new plastics observed another increase after their initial decrease from 11.5 nm, which was between midpoints 20.5 and 36.5 nm (PET new: +2400 particles/cm^3^; PP new: +18,000 particles/cm^3^; HDPE new: +1100 particles/cm^3^). Regardless, the concentrations measured at the 11.5 and 15.4 nm midpoints were 2–8× the amount of the next highest concentration.

In regard to the 0.3–10 μm particles quantified by the OPS, all six categories of plastic had the highest particle concentrations at the smallest diameter midpoint of 0.337 μm. While particle concentrations generally decreased after the maximum, there were two main increases: between midpoint 0.809 and 1.007 μm (PET waste: +130 particles/cm^3^; PP waste: +7 particles/cm^3^; HDPE waste: +90 particles/cm^3^; PET new: +9 particles/cm^3^; PP new: +15 particles/cm^3^; HDPE new: N/A) and between midpoint 1.254 and 1.944 μm (PET waste: +200 particles/cm^3^; PP waste: +14 particles/cm^3^; HDPE waste: +110 particles/cm^3^; PET new: +28 particles/cm^3^; PP new: +47 particles/cm^3^; HDPE new: +4 particles/cm^3^). Furthermore, during the shredding of new PP, the highest concentrations after the maximum were between midpoints 1.944 and 7.272 μm, which was much larger than what was observed for the other plastics. New PET also saw a similar trend, but it was not as prominent. However, the waste plastics in particular did see an increase at these larger size ranges whatsoever.

##### Imaged particle size and morphology

**Fig. 3:**
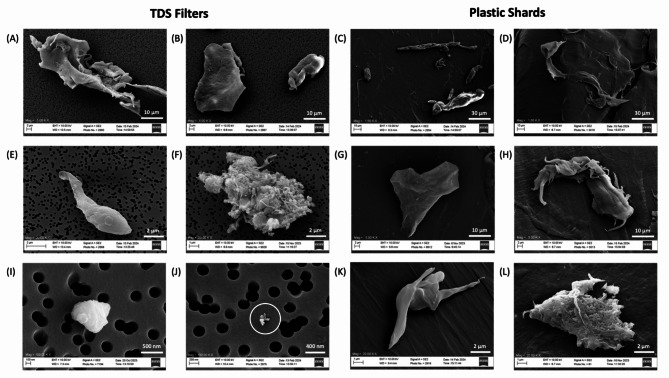
SEM imaging of the airborne particles collected on the TDS filter (A, B, E, F, I, J) and particles found on the plastic shard outputs released from the shredder (C, D, G, H, K, L). For the TDS filters, the following magnifications were used: 5,000× (A, B), 20,000× (E, F), and 100,000× (I, J). For the plastic shards, the following magnifications were used: 1500× (C, D), 5000× (G, H), and 20,000× (K, L). The images are not categorized by plastic type or origin because the variation in morphology is representative of what was observed across all categories of plastic.

Scanning electron microscope (SEM) images of the airborne particles collected on the TDS filter, as well as the plastic shard outputs released from the shredder, were taken to further assess particle size and morphology and are presented in Fig. 3. While there was a limitation in chemically confirming the presence of plastic during SEM imaging, we were able to verify the presence of plastic material because the plastic melted under the SEM electron beam. The high carbon component within the melting particles indicated a plastic composition. Moreover, only particles related to the shredding process, specifically those composed of plastic, were anticipated since the experiments took place in a cleanroom equipped with HEPA filter ventilation.

**Fig. 4:**
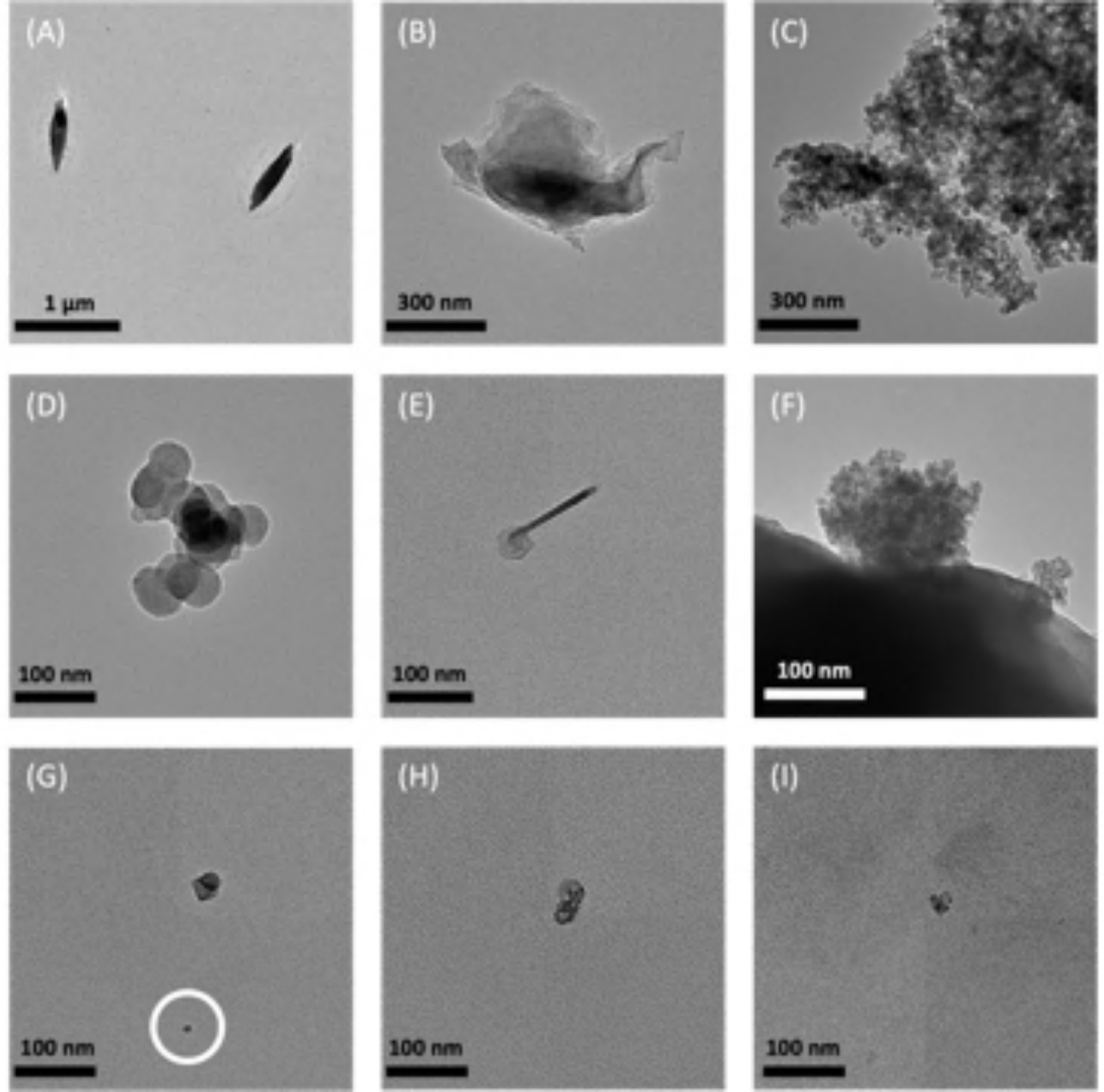
TEM imaging of airborne particles collected on the TDS grids. The magnifications are as follows: 21,000× (A-C) and 52,000× (D-I). The images are not categorized by plastic type or origin because the variation in morphology is representative of what was observed across all categories of plastic.

The sizes of the particles on the filter range from approximately 10–30 μm (A–B), 5–10 μm (E–F), and 200–600 nm (I–J). The sizes of the particles on the plastic shards range from approximately 10–100 μm (C–D), 30–50 μm (G-H), and 1–5 μm (K–L). We found that morphology varied drastically between particles and did not adhere to a consistent shape. Figure 4 is not categorized by plastic type or origin because the images included are representative of the variation in particle shape witnessed across all six categories of plastic. In general, particles over one micron had the largest differences in morphology and were typically twisted and warped into elaborate shapes. While the submicron particles, shown in Fig. 3I and J, consisted of much smaller fragments and were less complex than the larger particles, they too did not adhere to a specific shape. The images of the plastic shards similarly reveal that there is a clear diversity in the particles generated by shredding. We can reasonably conclude that shredding produces nonuniform particles that cannot be easily classified into defined shapes.

The particles collected on the TDS grid and imaged using a transmission electron microscope (TEM) are presented in Fig. 4. Similarly to the SEM, there was no way to confirm the presence of plastic during TEM imaging, which is a limitation of this research; however, the presence of plastic was indicated via the methods (melting under the direct bean of the microscope) described above.

The sizes of the particles shown range from approximately 0.6–10 μm (A–C), 50–150 nm (D–F), and 10–50 nm (G–I). Figure 4G–I corroborate the presence of smaller nanoparticles between 10 and 50 nm as detected by the SMPS. Similar to the SEM results, there was a great variation in morphology among particles. Accordingly, Fig. 4 is not categorized by plastic type or origin because the images included are representative of the variation in particle shape witnessed across all six categories of plastic.

However, while the particles imaged were not characteristic of any one shape, some patterns were noticed. Waste PP had several particles that were long and oval shaped, as shown in Fig. 4D, while new PP had several particles that had fiber-like structures with circular bulbs at the end, as demonstrated in Fig. 4H. Particles with clusters of smaller round fragments, like those displayed in Fig. 4F, G, and I, were observed for waste and new HDPE and new PET.

#### Elemental composition

**Fig. 5:**
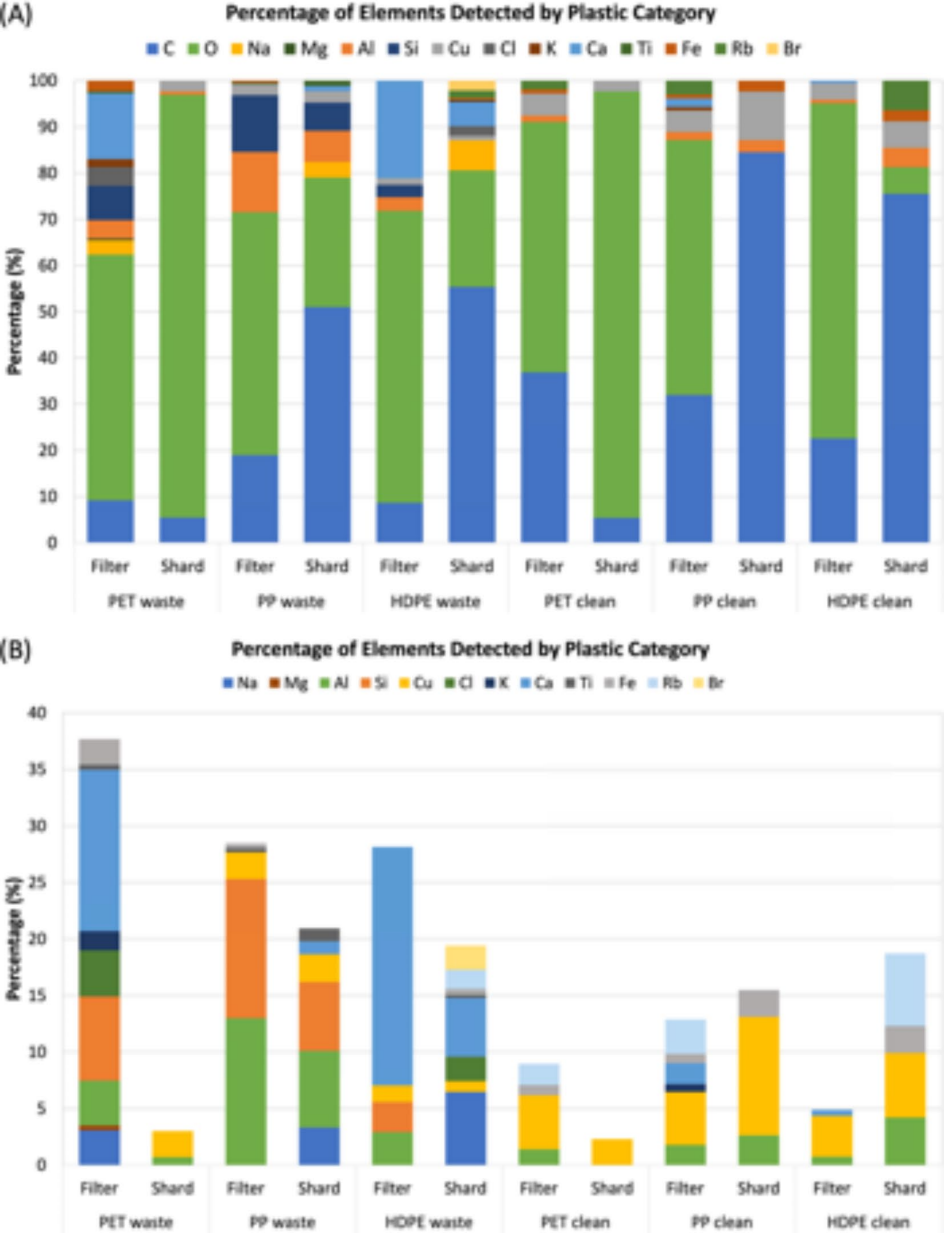
Elemental composition analysis of the airborne particles collected on the TDS filter and the plastic shard outputs released from the shredder using SEM/EDS. The figure provides a breakdown of (A) all elements detected excluding Au, and (B) the elements detected excluding C and O in order to understand what additional elements were present. The colors used in (A) and (B) are not consistent.

**Fig. 6:**
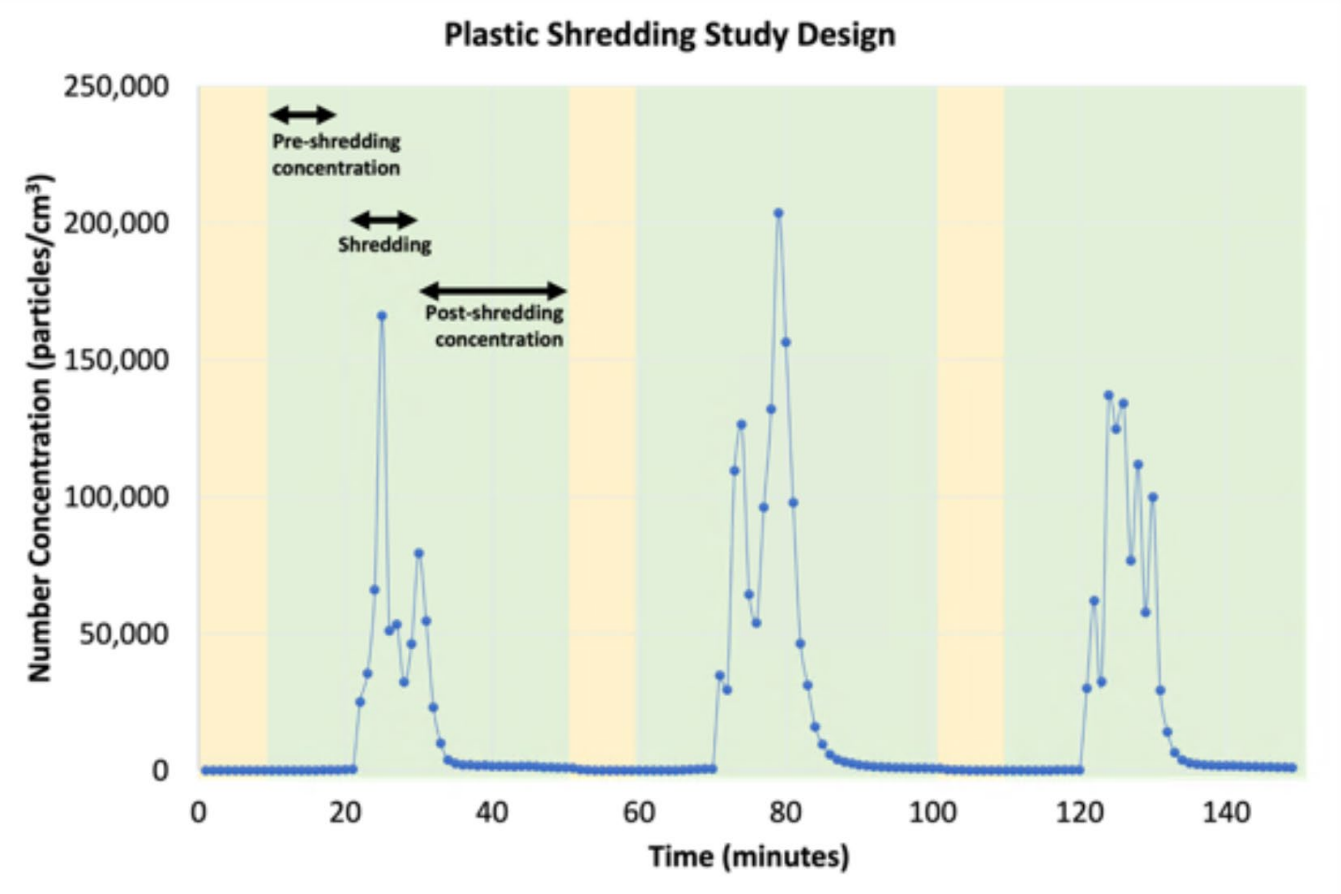
Study design used to shred plastic in the cleanroom of the lab. The periods highlighted in yellow represent times when the cleanroom ventilation was on. The periods highlighted in green represent times when the ventilation was off.

The chemical compositions of the particles collected on the TDS filters and the plastic shards were analyzed using SEM with energy dispersive X-ray spectrometry (SEM/EDS). The percentages of elements detected for each plastic are displayed in Fig. 6. For each plastic category, Fig. 5 presents one SEM/EDS sample from the airborne particles on the TDS filter and one SEM/EDS sample for the plastic shards. Furthermore, Fig. 5B shows the percentages of elements excluding carbon and oxygen to include a more detailed breakdown of the potential plastic additives or adsorbed chemicals.

SEM/EDS showed the presence of three main elements: gold, carbon, and oxygen. Carbon appeared across all SEM/EDS maps, which corresponds to the composition of plastic polymers. The chemical formulas of PET, PP, and HDPE are C_10_H_8_O_4_, C_3_H_6_, and (C_10_H_8_)_n_, respectively. The presence of plastic was also verified by the melting of the particles under the SEM/EDS electron beam due to the associated heat energy. In addition, only particles associated with the shredding process (i.e. those made of plastic) were expected because the experiments were conducted in a cleanroom with HEPA filter ventilation. Oxygen was also detected in all SEM/EDS maps. In addition to O being in the chemical formula of PET, it is commonly used in plastic production as a key element in plasticizers.

Na, Mg, Al, Si, Cu, Cl, K, Ca, Ti, Fe, Rb, and Br, which are referred to as additional elements, were also detected in the samples and are displayed in Fig. 5B. The presence of additional elements can in part be attributed to the numerous additives incorporated into plastics. For example, fillers, plasticizers, pigments, and foaming agents are added to improve plastic properties. Processing aids, lubricants, heat stabilizers, acid scavengers, and antistatic agents ease processing. Antioxidants, UV stabilizers, flame retardants, and antimicrobials are included to increase durability. Accordingly, the presence of other elements is expected.

Fillers frequently include Al, Si, Mg, Ca, and Ti, (in the form of clays, silica, glass, chalk, talc, alumina, and rutile), plasticizers can contain Cl, and colorants are usually sourced from metals, including Ti, Cu, Fe, Al, Cd, Zn, and Ni^30^. For example, titanium dioxide (rutile) is the most widely used white pigment^30^. Existing scientific literature has similar results as the findings herein. One study identified several heavy metals in the commercial additives found within PP and HDPE waste plastic, including Ni, Cd, Co, Cu, Pb, Mn, Cr, Zn, Fe^31^. Another study examined the elemental composition of PET, PE, and PP packaging waste streams in European sorting facilities and found the presence of C, H, O, Ti, Mg, Fe, Na, Al, Ca, Cl, and more^32^. It is important to note that Si, Cu, Cl, Ti, Fe, and Br can be hazardous at some doses and their concentrations in real facilities should be investigated further.

The waste plastics typically had more elements than their new counterparts. For the airborne particle samples, waste plastics consisted of 28–38% of additional elements while the new plastics consisted of 5–13% of additional elements. In regard to the plastic shard outputs released from the shredder, waste PP and HDPE shards again had higher concentrations of additional elements compared to new PP and HDPE shards (19–21% compared to 16–19%). However, for PET, both the waste and new shards had similar percentages of additional elements, 2–3%, which were the lowest out of all samples (waste PET was slightly higher).

As for the types of additional elements, Al and Cu were the most common across the six categories of plastic showing up in 10 and 11 of the 12 samples, respectively. Al had larger concentrations detected in the waste plastic (4.57% average in the waste samples compared to a 1.80% sample average in the new samples), while Cu surprisingly had higher concentrations in the new plastics (1.59% average in the waste samples versus a 5.26% sample average in the new plastics). While Al and Cu were present in the greatest number of samples, Ca was seen with the highest concentrations, 21% and 14% in the HDPE and PET filter samples, respectively, and had a 6.95% average across all waste samples. Next, while Si and Na were not detected in the new plastics, they were present in considerable amounts in the waste plastics (4.75% average Si concentration and 2.15% average Na concentration).

#### Zeta potential

Zeta potential was measured to assess the particles’ surface chemistry and agglomeration potential. In general, particles with a zeta potential between − 10 and + 10 millivolts (mV) are neutral, while those with zeta potentials greater than + 30 mV or less than − 30 mV are considered to have strong positive and negative charges, respectively^33^. When zeta potential is close to zero, also called the isoelectric point, particles tend to agglomerate in solutions^34^. Agglomeration and aggregation are known to occur at zeta potentials less than 5 mV^35,36^. On the other hand, strongly cationic or anionic particles have good physical stability and tend to repel each other such that no agglomeration occurs^34,35^.

The average zeta potential measurements of the airborne particles collected on the PVC filters for NMAM 500 for all categories of plastic, as well as the control filter, were between approximately − 2 and 2 mV, indicating that the particles are neutral. These findings provide evidence that the airborne particles generated by plastic shredding may agglomerate. The presence of agglomerates was further verified in the SEM and TEM imaging. However, while the findings herein suggest particle agglomeration, the SMPS data demonstrates that particles or their agglomerates were between 10 and 30 nm are most prominent. Regardless of whether these are individual particles or agglomerates, it is widely recognized that 10 to 30 nm particles have the highest deposition fraction in the alveoli and consequently can result in health impacts. As such, emissions during shredding should be adequately controlled for.

## Discussion

The rapid escalation of plastic production and consequent abundance of waste has resulted in the need to recycle our plastic goods. Plastic shredding is frequently used in mechanical recycling facilities and emits airborne particulates that have not been extensively examined in current scientific literature. Our findings suggest that such operations could expose workers to airborne MPs/NPs, in some scenarios to concentrations well over 1,000,000 particles/cm^3^, several orders above background level, especially those in the submicron range. In situ emissions may be higher than those observed in this study because mechanical recycling facilities continuously operate more than one industrial-grade shredder that are larger than the shredder used herein. Furthermore, while the data collected by the SMPS indicated that PP waste generated the highest level of submicron emissions, it is not practical to suggest that PP waste be avoided or not shred. PP is one of the most common thermoplastics and still needs to be recycled. As such, adequate engineering control measures and the use of personal protective equipment should be mandated during shredding activities.

Waste plastics consistently generated higher emissions than their new, unused counterparts, as well as consisted of a higher number of elements. There are several explanations for the increased emissions and higher number of elements detected in the waste plastic particles and shards: (1) the assortment of waste plastic had more adhesives, ink, and labels on the exterior of that plastic contributing to the presence of additional elements, (2) the waste plastics contained more additives compared to the new plastic, such as color (the new plastics sourced were clear or white), (3) each category of the new plastics consisted of only one or two different products while there was a much larger assortment of different kinds of waste plastic, (4) the waste plastic could adsorb chemicals from the environment, and (5) the waste plastic may have residual contamination from previous uses despite being cleaned.

In general, the elemental composition analysis found that Al, Cu, Ca, Si, and Na were the most common additional elements detected in the new and waste plastic samples. The presence of Al, Cu, Ca, Si, and Na in plastic can be linked to several different plastic additives. Al as alumina (Al_2_O_3_) is used in a popular flame retardant, as well as in stabilizers and pigments; Cu as a metal oxide is incorporated in antioxidants, color pigments, and biocides; Ca is combined with plastics as chalk (CaCO_3_) as an inexpensive filler to lower the cost of materials; and Si, in the form of silica (SiO_2_), is commonly used to enhance the handling, quality, and performance of plastic^10,28,30,31,37^. Lastly, Na can be present in plastic in compounds such as sodium benzoate (C_6_H_5_COONa) and sodium sulfate (Na_2_SO_4_), which work as clarifiers to improve the transparency of plastic products^28,38^.

There are several limitations of this study. First, while waste plastic was cleaned with warm water and soap and dried before being shred, contamination of the plastic waste from previous uses is an inherent risk. However, this also represented the practical shredding work involving various waste plastic somewhat contaminated. Second, as previously discussed, the particles imaged during SEM and TEM could use carbon element and the melting feature to verify as plastic. Although using carbon and melting feature is not a standard method, it’s resulted from scientific evidence based on the nature of plastic polymer material and our experiments. All experiments and particles collection occurred in the cleanroom of the lab with HEPA filter ventilation, resulting in the expectation for very little, if any, background particles. As such, the particles analyzed were anticipated to be associated with the shredding process of the plastics.

This study indicates that further research documenting the levels of airborne particulate matter present at mechanical recycling facilities is needed to bridge the gaps in the current body of scientific literature. Additionally, research exploring the potential health risks associated with the inhalation of high levels of nanoplastics between 10 and 30 nm generated during shredding should be explored. This is especially important because the deposition of particles in the lungs and cellular uptake of MPs/NPs produced during plastic shredding may be more difficult to generalize due to the lack of uniformity between the particles. Additionally, it is important to note that particles with rod-like features were identified in the SEM and TEM imaging. As previously discussed, research suggests that rod-like particles with larger aspect ratios can reach the deeper areas of the respiratory system and, thus, may pose a threat if inhaled during shredding.

## Methods

### Materials and study design

Both waste and new PET, PP, and HDPE were studied, for a total of six categories, in order to determine how emissions differed throughout stages of a plastic product’s life cycle. Plastic waste was collected from recycling bins on campus and residential apartments and washed to clean off food contamination. New, unused plastic of the same plastic type was purchased from vendors. For new PET and PP, clear cups were used. For new HDPE, opaque juice containers and white gallon jugs were used. All new, unused plastic that was ordered did not have logos or labels. The plastic was cut into smaller pieces with scissors or box cutters to fit into the mouth of the shredder and avoid obstructing the shaft. A 220 V heavy duty plastic shredder (INTBUYING, Rancho Cucamonga, CA, USA) was used to shred the plastic. The shredder was relatively small with the following dimensions: 70.1 × 89.9 × 104.9 centimeters (27.6 × 35.4 × 41.3 inches).

For each day of sampling, three experiments were conducted. The shredding of each plastic category occurred over at least three days (each with three individual experiments) to gather reliable and reproducible data. The experimental procedure was conducted in the cleanroom with a ventilation system and high efficiency particulate air (HEPA) filters, abbreviated to fans for the system. A filtration system was established in order to clear out the room for each experiment. The following steps, which are also displayed in Fig. 6, were taken:

1. Fans turned on to clear out the room until background concentration was zero.
2. Fans turned off for 10 min to measure baseline background concentration.
3. Shredding occurred for 10 min with fans still off.
4. Fans stayed off for 20 min to measure post-shredding concentration.
5. Fans turned on for 10 min to clear out room for the next experiment.
6. Fans turned off for 10 min for background concentration to return to the baseline level.
7. Repeat 10-minute shredding experiment and associated steps two more times for a total three experiments per day of sampling.

The time periods of baseline background concentration were used as control measurements which allowed us to compare the particulate emissions during shredding. The temperature and relative humidity in the cleanroom ranged from 294.15 to 307.15 K (21–34 °C) and 40–45%, respectively.

### Particle monitoring and sampling procedure

#### Direct reading devices

To measure particle concentration and size distribution, two direct reading devices were used: a NanoScan SMPS (Model 3910, TSI, Shoreview, MN, USA) which measures particle sizes from 10 to 420 nm and an OPS (Model 3330, TSI, Shoreview, MN, USA) which measures particles from 0.3 to 10 μm. The NanoScan SMPS was abbreviated to SMPS in this study. To evaluate the source emissions from plastic shredding, the devices were connected to conductive tubing that was fed to the mouth of the shredder.

#### Filter samples

To measure mass concentration and perform particle identification, a Tsai Diffusion Sampler (TDS) and NIOSH Manual of Analytical Methods (NMAM) 0500 and 0600 were used^39^. The TDS collects respirable and nanosized particles onto a 25-mm diameter polycarbonate (PC) filter, 0.22-µm pore size, with a carbon-coated 400-mesh TEM grid attached at the center which allows direct analysis of particles^40^. The TDS was connected via tubing to a GilAir Plus personal air sampling pump, which was set to a flow rate of 1 L/min. The operating flow rate was adjusted for a short-term sampling practice. Pre and post calibration of the air pump was conducted on a Mesa Labs DryCal Defender 510.

NMAM 0500, which is for the collection of particulates not otherwise regulated, involved the use of a tared 37-mm, 5-µm pore size polyvinyl chloride (PVC) filter inside of a cassette that operated at a flow rate of 1.5 L/min. NMAM 0600, which is for the collection of respirable particulates not otherwise regulated, involved the use of a 10-mm aluminum cyclone and same PVC filter, which was operated at a flow rate of 2.5 L/min. Both NMAN 0500 and 0600 utilize the same air pumps and calibration process as described for the TDS. Each filter substrate was weighed before and after sampling to determine the change in mass. Mass concentration (mg/m^3^) was calculated using the change in mass during the sampling duration with the set flow rate.

### Analytical methods

#### Microscopy

The particles collected on TDS filters and grids, as well as the plastic shard outputs released from the shredder, were analyzed with microscopy. Particles collected on the PC filters and the surfaces of the plastic shards were analyzed using SEM (Supra 40VP, Zeiss, Oberkochen, Germany). SEM images were taken at 10 kV beam voltage using a secondary electron (SE) detector at various levels of magnification. Prior to imaging, the filters and shards were placed on aluminum stubs with carbon tape and sputter coated with gold using a Pelco SC-7 (Pelco, Fresno, California, USA) for 60 s, which corresponds to approximately 3–4 nm of gold, in order to make the specimens electrically conductive for SEM. Particles collected on the grids were analyzed using TEM (Tecnai T12, FEI, Hillsboro, Oregon, USA). The TEM microscope was operated at 120 kV beam voltage at various levels of magnification. Microscopy allows for the imaging of substances in the micrometer to nanometer size range and consequent analysis of the morphological characteristics and sizes of the samples.

#### Elemental composition analysis

The chemical composition of the particles on the TDS filters and the plastic shards were analyzed using SEM/EDS. Using the SEM microscope detailed above, EDS was taken using the Dry Noran System Six EDS system at 20 kV beam voltage. Reference SEM images were taken at 20 kV with a SE detector. EDS maps were acquired in different areas of the sample(s).

#### Zeta potential

The zeta potential of particles collected on the PVC filters with NMAM 0500 was measured using a ZetaPALS (Brookhaven Instruments Corporation, Holtsville, NY). The filters were suspended in a solution of purified water and sonicated for 30–60 s in order to release the airborne particles. The filters were removed for each sample, and the solutions were then measured. In addition to the six types of plastic, the zeta potential of two controls were measured: an unused filter to control for the PVC filter and a blank control with no filter. However, the blank control with no filter resulted in a low detection signal and produced data that was not reliable and or included this study. Zeta potential measurements were gathered in order to identify the particles’ surface chemistry properties and analyze a particle’s tendency to agglomerate.

## Data Availability

All data generated or analyzed during this study are included in this published article and can be available from the corresponding author on reasonable request. The data sets are stored in the university BOX folder and accessible upon request.
